# An adaptable but threatened big cat: density, diet and prey selection of the Indochinese leopard (*Panthera pardus delacouri*) in eastern Cambodia

**DOI:** 10.1098/rsos.171187

**Published:** 2018-02-07

**Authors:** Susana Rostro-García, Jan F. Kamler, Rachel Crouthers, Keo Sopheak, Sovanna Prum, Visattha In, Chanratana Pin, Anthony Caragiulo, David W. Macdonald

**Affiliations:** 1 Wildlife Conservation Research Unit, The Recanati-Kaplan Centre, Department of Zoology, University of Oxford, Tubney House, Abingdon Road, Tubney, Abingdon OX13 5QL, UK; 2 Panthera, 8 West 40th Street, 18th Floor, New York, NY 10018, USA; 3 World Wide Fund for Nature Cambodia, Street 352, Boeun Keng Kang I, Phnom Penh, Cambodia; 4 Provincial Department of Environment, Mondulkiri, Sen Monorom, Cambodia; 5 Sackler Institute for Comparative Genomics, American Museum of Natural History, 79th Street at Central Park West, New York, NY 10024, USA

**Keywords:** *Bos javanicus*, food habits, intersexual differences, *Panthera pardus*, population decline, spatially explicit capture–recapture models

## Abstract

We studied the Indochinese leopard (*Panthera pardus delacouri*) in eastern Cambodia, in one of the few potentially remaining viable populations in Southeast Asia. The aims were to determine the: (i) current leopard density in Srepok Wildlife Sanctuary (SWS) and (ii) diet, prey selection and predation impact of leopard in SWS. The density, estimated using spatially explicit capture–recapture models, was 1.0 leopard/100 km^2^, 72% lower than an estimate from 2009 at the same site, and one of the lowest densities ever reported in Asia. Dietary analysis of 73 DNA confirmed scats showed leopard consumed 13 prey species, although ungulates comprised 87% of the biomass consumed (BC). The overall main prey (42% BC) was banteng (*Bos javanicus*), making this the only known leopard population whose main prey had adult weight greater than 500 kg. Consumption of wild pig (*Sus scrofa*) was also one of the highest ever reported (22% BC), indicating leopard consistently predated on ungulates with some of the largest adult weights in SWS. There were important differences in diet and prey selection between sexes, as males consumed mostly banteng (62% BC) in proportion to availability, but few muntjac (*Muntiacus vaginalis*; 7% BC), whereas females selectively consumed muntjac (56% BC) and avoided banteng (less than 1% BC). Predation impact was low (0.5–3.2% of populations) for the three ungulate species consumed. We conclude that the Indochinese leopard is an important apex predator in SWS, but this unique population is declining at an alarming rate and will soon be eradicated unless effective protection is provided.

## 1. Introduction

The leopard (*Panthera pardus*) has the widest distribution of any felid species, owing to its ability to inhabit diverse habitats ranging from tropical forests and savannahs, to deserts and boreal forests [1]. The leopard also has the broadest diet of all felids, and of all large carnivores, ranging in size from insects to giraffes (*Giraffa* spp.) [2]. Despite its adaptability in habitat use and diet, the leopard has experienced severe declines in distribution and numbers, occupying only 25–37% of its historical range [3], which led to its recent uplisting to Vulnerable by the IUCN [1]. The primary reasons for the decline are habitat loss, prey declines, conflict with humans and poaching for the wildlife trade, with the relative importance of these factors varying among regions [1,3]. Consequently, the leopard now occurs in mostly small and fragmented populations, especially in Asia where seven of eight subspecies are either already listed, or are being petitioned to be listed, as Endangered or Critically Endangered [3–5].

The Indochinese leopard (*P. pardus delacouri*) is a genetically distinct subspecies [6–8] that historically occurred throughout all mainland Southeast Asian countries and southeastern China. However, a recent review showed the Indochinese leopard likely now occurs only in 6.2% of its historical range [5]. The Indochinese leopard is extirpated in Singapore, likely extirpated in Laos and Vietnam, nearly extirpated in Cambodia and China, and has greatly reduced distributions in Malaysia, Myanmar and Thailand [5]. Rostro-García *et al*. [5] identified three priority sites and strongholds for leopard conservation in Southeast Asia: (i) the Northern Tenasserim Forest Complex on the Thailand–Myanmar border, (ii) Peninsular Malaysia and (iii) eastern Cambodia.

In Cambodia, the Indochinese leopard has declined dramatically, and now occurs only in 8% of its historical range in the country [5]. The only potentially viable population remaining in the country occurs in the Eastern Plains Landscape (EPL) [5], a large protected-area complex covering greater than 10 000 km^2^ in eastern Cambodia [9]. In contrast to most of Southeast Asia, the EPL is dominated by savannah-like habitat (i.e. dry open deciduous forests), and is unique for containing the last remaining population of pure spotted leopard in Southeast Asia (all other leopard populations in Southeast Asia appear to contain only melanistic leopard, or a mixture of melanistic and spotted leopard) [5]. In 2009, the leopard density within one protected area of the EPL, Srepok Wildlife Sanctuary (SWS), was estimated to be 3.6 leopard/100 km^2^ [9], indicating this landscape likely contained a relatively high leopard population. However, increased levels of poaching that have led to rapid declines of tiger and leopard populations throughout Southeast Asia [5,10–12] might have led to a decline of the leopard population in EPL, although the extent of the possible decline is unknown.

The EPL is known for its high biodiversity, and for having the world's largest population of endangered banteng (*Bos javanicus*) [13], as well as populations of several other threatened ungulate species, such as Eld's deer (*Rucervus eldii*) [14]. The EPL also has a diverse community of at least seven primate species [15], six of which are classified as threatened by the IUCN. The few dietary studies of the Indochinese leopard conducted in evergreen forests in Thailand showed that it preys mainly on relatively small species, such as primates, small ungulates and small carnivores [16–18]. In contrast to Thailand, the EPL is dominated by dry open forest which supports a higher ungulate biomass [14] and presumably lower primate density. However, the extent to which leopard use the potential prey species in this habitat type is unknown, although such information is important to provide insight into the predatory niche of leopard in the community, and determine whether leopard predation negatively impacts threatened prey populations.

Male leopard is 30–50% larger in body mass than female [19], similar to the sex differences found in other large felids, which may result in inter-sexual differences in diet and prey selection. For example, male cheetah (*Acinonyx jubatus*) and male cougar (*Puma concolor*) were shown to prey on larger ungulate species than females [20–22]. However, such sex-specific differences have not yet been investigated for leopard in Asia.

The purpose of this study was to investigate the ecology of the last potentially viable population of Indochinese leopard in Cambodia, at a globally important priority site for the subspecies. The specific objectives were to: estimate the density of leopard; determine the diet and prey selection of leopard, with a focus on differences between males and females; and determine predation impact of leopard on the community of threatened ungulate species in SWS, the largest protected area within the EPL. We predicted that: (i) the leopard density would have decreased, owing to recent increases in poaching in the region; (ii) leopard diet would be dominated by primates, small (less than 30 kg) ungulates and other small prey; (iii) leopard would selectively consume prey weighing 10–40 kg, given its preferred prey range [2]; (iv) in general, male and female leopard would consume similar-sized prey species; and (v) predation impact would be higher for small ungulate species. In order to meet the objectives, we used DNA confirmed leopard scats, camera-trap and line-transect data from SWS.

## 2. Material and methods

### 2.1. Study area

The SWS (3725 km^2^), formerly named Mondulkiri Protection Forest up to 2016, is located in eastern Cambodia within the EPL, a complex of protected areas covering greater than 10 000 km^2^ [9]. A core zone (approx. 1700 km^2^) is located in the eastern part of SWS. There are five ranger stations located throughout SWS that are permanently manned by rangers, three of which are within or bordering the core zone, in addition to several substations that are manned by rangers periodically. No villages occur within SWS core zone, although several villages occur along the northern, western and southern areas of SWS. Local villagers are allowed to enter SWS to collect non-timber forest products, graze cattle, fish and hunt unprotected species for subsistence using traditional methods.

The habitat of SWS is dominated by open dry deciduous dipterocarp forests, interspersed with smaller patches of mixed deciduous, evergreen, semi-evergreen and riverine forests. The SWS has a distinct dry season for approximately half the year (December to May), followed by a rainy season (June to November). There are seven species of ungulates in SWS, several of which are classified as Threatened (IUCN classification follows each name), including banteng (EN), Eld's deer (EN), wild water buffalo (*Bubalus arnee*—EN), sambar (*Rusa unicolor*—VU), gaur (*Bos gaurus*—VU), Eurasian wild pig (*Sus scrofa*—LC; hereafter wild pig) and red muntjac (*Muntiacus vaginalis*—LC; hereafter muntjac) [14]. There are at least seven primate species in EPL [15], which include the red-cheeked gibbon (*Nomascus gabriellae*—EN), stump-tailed macaque (*Macaca arctoides*—VU), pygmy slow loris (*Nycticebus pygmaeus*—VU), black-shanked douc (*Pygathrix nigripes*—EN), long-tailed macaque (*Macaca fascicularis*—LC), northern pig-tailed macaque (*Macaca leonina*—VU) and Indochinese lutung (*Trachypithecus germaini*—EN), the latter five species having been confirmed in SWS. The tiger was recently extirpated in EPL [23], and in SWS the last tiger photograph and paw print were recorded in 2007 and 2009, respectively. Other felid species recorded in EPL that potentially occur in SWS include Asiatic golden cat (*Catopuma temminckii*—NT), clouded leopard (*Neofelis nebulosa*—VU), jungle cat (*Felis chaus*—LC), leopard cat (*Prionailurus bengalensis*—LC) and marbled cat (*Pardofelis marmorata*—NT). Other carnivore species include Asiatic jackal (*Canis aureus—*LC), dhole (*Cuon alpinus*—EN) and at least 10 species of smaller carnivores [24].

### 2.2. Camera trapping and density estimation

**Figure 1.**
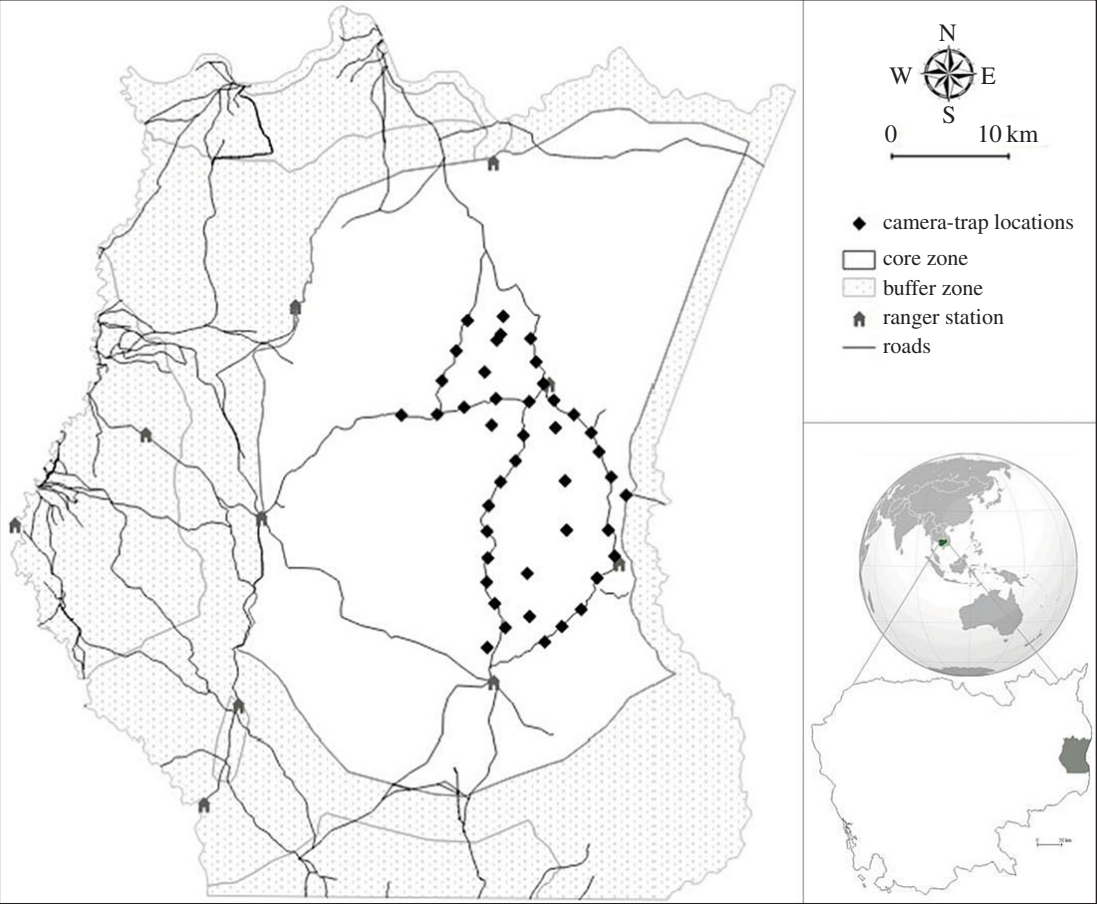
Map of SWS, Cambodia, indicating the camera-trap locations (*n* = 42) used to estimate the Indochinese leopard (*Panthera pardus delacouri*) density in 2014. The inset on the right shows the location of SWS in Cambodia.

In order to compare our survey results to those obtained in 2009, we followed the same field methodologies reported by Gray & Prum [9]. Consequently, we used the same camera traps (Reconyx RapidFire Professional PC90; Reconyx, Inc., Holmen, WI, USA) during the same season (i.e. dry season) and placed them in pairs along the same roads and trails, with the same spacing (2–3 km) between camera stations [9]. Cameras were placed on trees located 2–3 m from the middle of the trail, and motion beams set to trigger at a height of 40–45 cm above the centre of the trail. We extended the area surveyed by Gray & Prum [9] (from 210 km^2^ to approx. 270 km^2^) to include roads and trails to the north and northwest (figure 1), in an attempt to record additional leopard, based on signs we observed. We used 42 paired camera stations that were set out in two blocks (each with 21 locations) due to the limited number of cameras (*n* = 42). We camera trapped the northern block from 18 February to 9 April 2014, and the southern block from 9 April to 3 June 2014. Cameras were operational for 24 h during the sampling sessions, and checked approximately halfway through each session within a block to change batteries and remove vegetation.

Two of the authors independently identified individual leopard based on its unique pelage markings, and any discrepancies were jointly reviewed to reach a final agreement on identification. Because leopard has low detection probabilities, individual capture histories were developed considering a 48-h period as one occasion, such that one encounter occasion consisted of a 2-day sampling period, and a binary matrix of leopard captures was constructed (i.e. detection history of individuals collated). The data were collapsed in this manner to avoid estimated detection rates close to 0 (photographic detections were sparse), as this can lead to estimation problems [25].

The demographic parameters of wild Indochinese leopard are unknown. As a consequence, it is difficult to estimate the time frame that adequately approximates a closed population. Relatively few tests for population closure have been developed in traditional capture–recapture [26,27], mostly because violation of demographic closure can be indistinguishable from behavioural variation in detection [28]. In addition, closure tests are limited in obtaining reliable estimates when small sample sizes are used [29]. In general, studies that focus on elusive species, such as the Indochinese leopard, have low sample sizes, and thus these tests might not be appropriate for revealing robust values [30]. Similarly, no specific population closure test for spatially explicit capture–recapture (SECR) models is available, given that violation of population closure cannot be distinguished from the violation of other model assumptions [31]. In the absence of suitable closure tests, a survey period of 2–3 months was suggested to be appropriate for the study of felids [30,32], including leopard (93 days) [33]. We used a 98-day subset of our data to estimate leopard density because we deemed this a period that would provide sufficient data for a species that has low capture probability, while ensuring demographic closure and minimizing the likelihood of activity centres changing considerably.

In order to compare the results with those obtained by Gray & Prum [9], density was estimated using SECR models using the maximum-likelihood estimator DENSITY (v. 5.0). Following Gray & Prum [9], we set a buffer width around the trapping grid of 10 km, assumed a half-normal spatial capture probability function and a Poisson distribution of home-range centres. In addition, leopard density was estimated using a 20 and 30 km buffer around the trapping grid (electronic supplementary material, table S1).

Bayesian inferences, unlike likelihood based procedure, do not rely on asymptotic arguments and are valid irrespective of sample size [34]. Therefore, given the relatively low number of both individuals and recaptures, we also estimated density using the Bayesian estimator SPACECAP (v. 1.1.0) [35] of R (v. 3.2.3) [36]. This spatial estimator requires three input files: (i) a trap deployment file; (ii) a detection history or capture file; and (iii) potential home-range centre file (demarcating potential home-range centre of leopard within suitable leopard habitat). For Bayesian models, we generated a 15 and 30 km buffer around a grid of equally spaced points (580 m intervals; each representing 0.336 km^2^), containing the trap array, to represent the probable leopard activity centres [34,37]. Because the leopard is a habitat generalist and the core area was surrounded by forested areas, we considered the totality of the buffer area as suitable habitat, and therefore the activity centres were assumed to be uniformly distributed over this space. In addition, during the dry season there is no body of water that could impede the movement of leopard within the landscape. We used 70 000 Markov chain Monte Carlo (MCMC) iterations, with the initial 20 000 values discarded during the analysis (burn-in period), and the data augmentation value was set to 10–30 times the number of photographed individuals. Models were run considering a global behavioural response (trap response absent), and fitted using a half-normal detection function and Bernoulli encounter model. The fit of each model was evaluated using the Bayesian *p* value [38], where extreme values (near 0 or 1) indicated a lack of model adequacy, whereas values near 0.50 indicated model adequacy. Model parameters were adjusted until convergence was adequate (Geweke z-scores between −1.6 and +1.6), Bayesian *p* values indicated model adequacy, and data augmentation, state space extent and sample size were sufficiently large.

### 2.3. DNA analyses of scats

During the dry seasons of 2013 and 2014, scats (i.e. faeces) were collected along 30 transects (2 km each) established on roads throughout the core zone of SWS, as well as opportunistically when conducting other research. For each scat, maximum diameter was measured (unless the scat was deteriorated) and GPS location was recorded. Approximately 10 g of each scat was stored in small paper envelopes, and then sent to the Sackler Institute for Comparative Genomics, American Museum of Natural History, for genetic analysis to determine species, individual, and sex. For species determination, genomic DNA was extracted and species were identified from scat as described in Caragiulo *et al*. [39]. Scat samples were identified to species by amplifying regions of four mitochondrial genes. PCR amplicons were sequenced as described in Caragiulo *et al*. [39] using the thermocycler profile of Platt *et al*. [40]. We purified sequencing amplifications via ethanol precipitation and sequenced them in an ABI 3730xl DNA Analyser (Applied Biosystems, Carlsbad, CA, USA). We manually edited sequences using Geneious v. 8.0 (www.geneious.com) [41], and compared them to both an in-house database of carnivore mtDNA sequences and the NCBI nucleotide BLAST database [42] to confirm species identification.

All scat samples identified as leopard were processed for individual and sex identification. Leopard samples were amplified using 12 microsatellite loci to identify individuals as described in Caragiulo *et al*. [43]. The sex of each sample was determined by amplifying portions of the zinc finger on the X and Y chromosomes [44] using the protocol described in Caragiulo *et al*. [43]. Each amplification was independently repeated a minimum of four times for both individual and sex identification (see Caragiulo *et al*. [43] for more details).

### 2.4. Diet and prey selection

The diet of leopard was determined by the analysis of the confirmed leopard scats, after washing them in a laboratory and drying the remains. For each scat, we separated hairs, claws, hoofs and bones from different prey species, and estimated per cent volume of each species. Prey items that were 1% or less of scats were excluded from analysis. Hair samples were identified to species by examining the structure of the medullas, which became visible under the microscope after soaking hairs in xylene for 24 h. Medulla structures were compared to those in a reference collection of hairs from known species. Hairs of leopard were found in some scats, although in trace amounts (≤5%), and these were excluded from analysis because we assumed they were ingested while grooming.

Results from scat analysis were quantified in terms of frequency of occurrence (i.e. percentage of scats containing a particular food item) for comparison purposes [46]. However, because the frequency of occurrence can be misleading given that smaller prey species contribute more to a scat than larger species [45,46], we obtained the per cent biomass consumed (BC). The per cent BC, considered the best approximation of the true diet [46], was calculated using a regression equation derived from feeding trials on captive cougar [47]. Cougar is similar to leopard in body size and niche, thus we assumed they would have a similar prey spectrum and therefore the model would be applicable to leopard [46], and this method has been used by previous studies to estimate leopard diet [45,48–52]. In the regression equation, *Y_i_* = 1.98 + 0.035*X_i_*, *X* is the live body mass of prey, whereas *Y* is the mass of prey per collected scat (*i*). The mean live body mass of all prey species was taken from Francis [53]. For all species less than 30 kg, we used the adult weight, or the mean of adult males or females if both were given. If we could not distinguish hairs between two species (e.g. black-shanked douc and Indochinese lutung), then we took the mean of weights given for those two species. For ungulates greater than 30 kg (i.e. banteng and wild pig) we used half the weight given for adult females, assuming leopard killed both adult females and young. We excluded arthropods and unknown mammal (total three scats) from the biomass calculations because we were unable to accurately estimate BC for these prey items.

We calculated Jacobs' index *D* [54]: *D* = (*r *– *p*)/(*r* + *p − *2*rp*), where *r* is the fraction of prey used, and *p* is the fraction of prey available. Jacobs’ indices were calculated to investigate prey selection using biomass data to assess which prey species were selected (0 < *D* ≤ 1) and which were avoided (−1 ≤ *D* < 0). For each prey species, the *D*-value depends on which other species are included in the calculation; therefore, we calculated *D*-values only for the three ungulate species that were consumed by leopard. To determine fraction of prey available, we used ungulate densities estimated by WWF Cambodia personnel. Ungulate densities were estimated using distance-based line transect sampling [55], following the same field protocols and transects of Gray *et al*. [14]. During the dry season of 2014, a total of 38 transects (2–3 km in length) were surveyed four to eight times within the SWS core zone (approx. 1700 km^2^). A total of 603 km was walked across all transects, with observers walking transects just after sunrise (start time = 05.00–07.30) and just prior to sunset (start time = 15.30–17.30). Data recorded for ungulates included: species; number of animals (cluster size); distance between the animal or centre of animal group and the observers (using laser rangefinders); compass bearing to the animal or animal group; and compass bearing of the transect line. Data were analysed using the program DISTANCE v. 6.2 [56]. In order to fit detection functions, number of encounters per species were pooled across two adjacent protected areas (SWS and Phnom Prich Wildlife Sanctuary (PPWS)) and densities were calculated only for those ungulates that had the minimum of 40 observations [55] (see Gray *et al*. [14] for more details). Species data were pooled from 2010, 2011 and 2014 to produce a global detection function. Densities were then stratified by protected area and year to produce ungulate densities for SWS. Biomass available for each ungulate species was obtained by multiplying the density estimates by the adult female weights given by Francis [53].

We calculated leopard diet and *D*-values based on all confirmed leopard scats that were collected, and then calculated diets and *D*-values for males and females separately based on the subset of scats that were assigned to sex. For scats assigned to different individuals, we calculated and compared the diet of individuals with ≥5 scats for illustrative purposes only.

We calculated predation impact on ungulates from an equation used previously for carnivores [57,58]: *N*_prey_ = (*D* × DFI × *B*_prey_ × *n*_days_ × 100)/BM_prey_, where *N*_prey_ is number of prey individuals consumed by leopard/100 km^2^, *D* is density of leopard/km^2^, DFI the daily food intake of leopard, *B*_prey_ is the per cent BC by leopard for a given prey species, *n*_days_ the number of days (i.e. 180 days representing the dry season), and BM_prey_ the mean live body mass of prey. The DFI for leopard was assumed to be 4.01 kg, based on the average of four different studies that calculated DFI for leopard [52,59–61]. The BM_prey_ was taken from Francis [53] (see above). For each ungulate species, *N*_prey_ was divided by density (individual/100 km^2^) to determine the per cent of ungulate population consumed by leopard. Predation impact could only be calculated for the 6-month dry season, as this was the only season we collected scats. Scats collected from two consecutive dry seasons were pooled in analysis.

## 3. Results

From 2147 trap days, we obtained a total of 26 independent events from 8 confirmed different leopards. Six leopards were photographed on both flanks, two leopards were photographed only on their right flank and one leopard was photographed only on its left flank, thus the total number of leopard was eight or nine. For analyses, we assumed eight different leopards were photographed (two females, two males and four of unknown sex). Given that some individuals were captured more than once by the same camera trap within a 48-h period, a total of 23 capture events were used in subsequent analyses. Using maximum-likelihood inference, the estimated *D* (± s.e.) was 1.0 ± 0.4 leopard/100 km^2^, which was 72% lower than the density of 3.6 ± 1.0 leopard/100 km^2^ estimated from 2009 using the same analysis [9]. The density estimate was the same regardless of whether we used a 10, 20 or 30 km buffer (electronic supplementary material, table S1). Using Bayesian inference, the estimate (posterior mean) of leopard density was of 1.0 leopard/100 km^2^ (post. s.d. = 0.4; electronic supplementary material, table S2), with a 95% posterior interval of (0.4, 1.6).

Six ungulate species were recorded during the line-transect surveys in SWS and PPWS, which included muntjac (*n* = 115), wild pig (*n* = 58), banteng (*n* = 44), gaur (*n* = 5), Eld's deer (*n* = 5) and sambar (*n* = 1). Mean (± s.e.) densities were 2.1 ± 0.3 muntjac km^−2^, 6.5 ± 2.0 wild pig km^−2^ and 2.2 ± 0.5 banteng km^−2^.

A total of 113 putative leopard scats was collected, and 73 were confirmed by genetic analysis to be leopard. Of the 40 scats not confirmed to be from leopard, 23 failed to detect any species, 9 were identified as dhole, 3 were identified as jackal or dog, 3 were identified as prey and 2 detected multiple carnivore species. Individual assignment was possible for 44 of the 73 confirmed leopard scats. When considering only scats that were collected from within the camera trapping grid during our survey period in 2014, nine different leopards were identified (four males, three females, two unknown), seven of which had greater than one scat collected. The nine different leopards identified from scats correspond to the maximum number of individuals identified from the camera-trap photos.

Sex could be assigned to 43 (23 males, 20 females) of the 73 confirmed leopard scats. Mean diameter (±s.d.) of confirmed leopard scats was 3.1 ± 0.4 cm (range: 2.1–4.0; *n* = 53), with a mean diameter of confirmed male scats of 3.2 ± 0.4 cm (range: 2.6–4.0 cm; *n* = 15) and of confirmed female scats of 2.9 ± 0.3 cm (range: 2.4–3.3 cm; *n* = 15).

**Figure 2.**
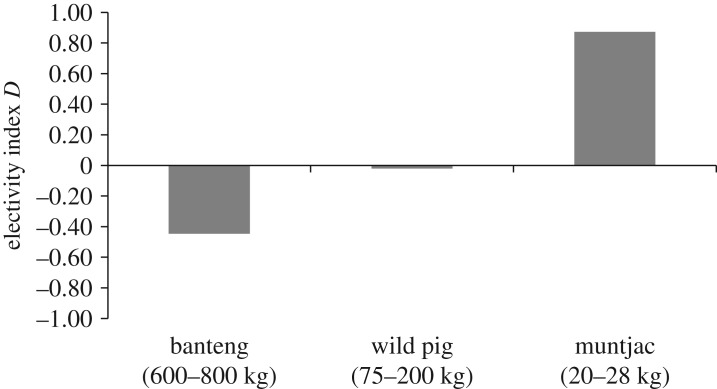
Jacob's electivity index (*D*) of the biomass of ungulates consumed by the Indochinese leopard (*Panthera pardus delacouri*) in SWS, Cambodia.

The 73 leopard scats contained a total of 103 items, comprising 13 prey species, ranging in size from insect to banteng (table 1). A total of 63.0% of scats contained one prey item, whereas 32.9% of scats contained two prey items, and 4.1% of scats contained three prey items. Ungulates comprised 86.5% of BC, followed by Malayan porcupine (*Hystrix brachyuran* hereafter porcupine, 4.3% BC), small carnivores (3.7% BC) and primates (3.5% BC; table 1). Banteng was the most consumed ungulate (42.2% BC), followed by muntjac (22.1% BC) and wild pig (22.1% BC; table 1). The biomass of ungulates consumed during the dry season did not reflect the biomass available, as leopard showed a strong positive selection for muntjac (*D* = 0.87), non-selection for wild pig (*D* = −0.02) and negative selection for banteng (*D* = −0.45; figure 2). Leopard consumed 6.7 individual muntjac/100 km^2^ (3.2% of the muntjac population), 4.3 individual wild pig/100 km^2^ (0.7%) and 1.0 individual banteng/100 km^2^ (0.5%) during the dry season.

**Table 1.** Diet of the Indochinese leopard, expressed as per cent biomass consumed (% BC) and frequency of occurrence per scat (% FOS), in SWS, Cambodia. All scats included were confirmed by genetic analysis to be from leopard, whereas male and female scats were a subset that was assigned to sex by genetic analysis.

prey category	all scats (*n* = 73)		male (*n* = 23)		female (*n* = 20)	
species	% BC	% FOS	% BC	% FOS	% BC	% FOS
ungulate	86.5	79.5	84.7	73.9	87.4	95.0
banteng (*Bos javanicus*)	42.2	17.8	61.5	30.4	0.5	5.0
wild pig (*Sus scrofa*)	22.1	39.7	16.6	43.5	31.3	30.0
muntjac (*Muntiacus vaginalis*)	22.1	38.4	6.5	17.4	55.7	70.0
carnivore	3.7	9.6	2.5	8.7	3.7	5.0
small Indian civet (*Viverricula indica*)	0.7	1.4	0	0	0	0
common palm civet (*Paradoxurus hermaphroditus*)	2.2	6.9	2.5	8.7	0	0
leopard cat (*Prionailurus bengalensis*)	0.7	1.4	0	0	3.7	5.0
primate	3.5	8.2	4.9	13.0	3.9	10.0
macaque (*Macaca* spp.)	1.6	4.1	3.0	8.7	2.1	5.0
Colobinae (*Pygathrix nigripes* and/ or *Trachypithecus germaini*)	1.9	4.1	1.9	4.4	1.8	5.0
others						
porcupine (*Hystrix brachyura*)	4.3	9.6	6.2	17.4	1.6	5.0
Burmese hare (*Lepus peguensis*)	1.4	4.1	1.7	4.4	3.4	10.0
small (less than 1 kg) rodent	0.8	2.7	0	0	0	0
unknown mammal	—	1.4	0	0	0	0
fresh-water crab (Potamidae)	—	1.4	0	0	0	0
termite	—	1.4	0	0	0	0

**Figure 3.**
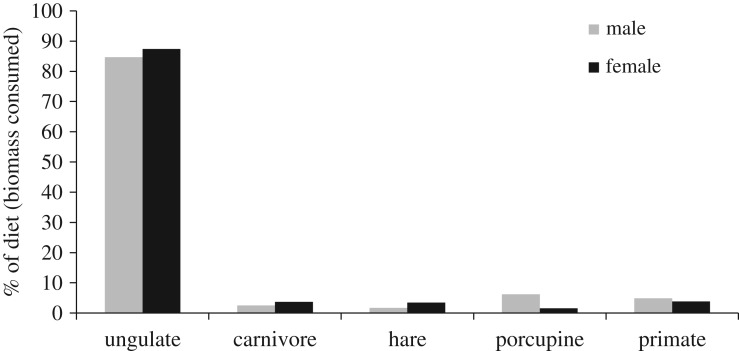
Diet of male and female Indochinese leopard (*Panthera pardus delacouri*) based on BC of five main prey categories in SWS, Cambodia.

**Figure 4.**
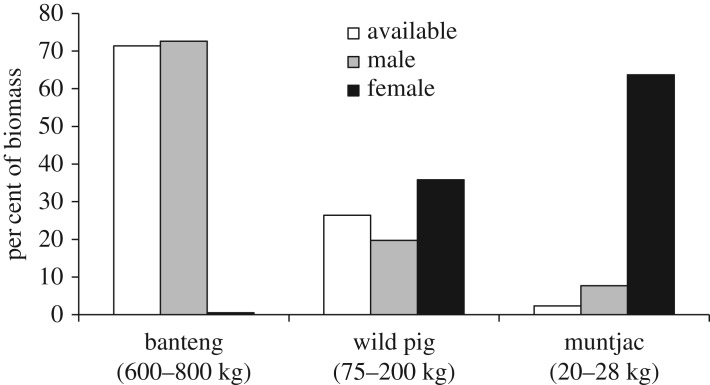
Biomass of ungulates consumed by male and female Indochinese leopard (*Panthera pardus delacouri*) compared to biomass available in SWS, Cambodia.

When diets were analysed separately by sex, the results showed that male and female leopard had similar diets across five broad prey categories (table 1; figure 3). Male and female diets were comprised mainly of ungulates (84.7 and 87.4% BC, respectively), whereas consumption of the other prey categories was less than 7% each (table 1; figure 3). However, when ungulate species were analysed separately, male and female leopard had opposite trends in consumption with regard to ungulate body size (figure 4). Male leopard consumed mainly banteng (61.5% BC) which was nearly 10 times the amount of muntjac consumed (6.5% BC; figure 4). In contrast, female leopard consumed mainly muntjac (55.7% BC) and had only a trace amount of banteng in its diet (less than 1% BC; figure 4). Male and female leopard consumed wild pig in moderate amounts (16.6% and 31.3%, respectively; figure 4). When compared with availability, consumption of all three ungulate species by male leopard was approximately in proportion to their biomass available (figure 4). By contrast, female leopard exhibited a strong positive selection for muntjac (*D* = 0.97) and a strong negative selection for banteng (*D* = −0.99; figure 4).

The diets of three male leopard hinted at individual specializations. One male (*n* = 7 scats) consumed mostly banteng (71.5% BC), a second male (*n* = 6 scats) consumed less banteng (40.1%) and more small (less than 10 kg) species, whereas a third male (*n* = 5 scats) did not consume banteng, and instead consumed mostly wild pig (50.0%) and porcupine (34.3%).

## 4. Discussion

The density of 1.0 leopard/100 km^2^ in SWS was one of the lowest densities reported in Asia. Previous studies using SECR models in Malaysia, Bhutan, Nepal and India showed leopard densities typically were 3–5 leopard/100 km^2^ [33,62–64], even in unprotected, human-dominated landscapes [65], and up to 9–13 leopard/100 km^2^ [66–68]. Only a population of the Critically Endangered Amur leopard was found to have similarly low densities of 1 leopard/100 km^2^ [69]. In our study, both methods for estimating density showed nearly identical results, and the camera-trap data showed a similar minimum number of individuals within the camera-trapping grid as the DNA analysis of scats (eight or nine leopards and nine leopards, respectively), suggesting our density estimate for the core zone of SWS was reliable.

The 72% decrease of leopard density in the core zone of SWS from 2009 to 2014 was not likely due to methodologies, because we used the same analysis and followed the same field protocols as the study in 2009 [9]. Similarly, the density decline of leopard was not likely due to prey declines, because densities of banteng, wild pig and muntjac remained stable or increased in SWS from 2009–2011 to 2014 [14]. Although we predicted that the leopard density would decline due to regional leopard declines and recent increases in poaching, we did not expect such a large decline. This is one of the largest density decreases recorded in a leopard population, and suggests the leopard population in SWS is rapidly heading towards extinction. Only two previous studies found a greater than 60% decrease in a leopard density in less than 6 years, which was attributed to an increasing density of reintroduced tiger inside protected areas in India [50,70].

Reasons for the rapid decline of leopard in SWS since 2009 are likely due to increases in poaching, particularly indiscriminate snaring. Across Southeast Asia, deforestation rates have increased recently to become the highest in the world, which has coincided with an explosion in the illegal wildlife trade fuelled by increased demand, thereby causing serious declines in many wildlife species [10,11,71], including leopard [5]. Demand is often highest for those species used in traditional Asian medicine such as tigers, and consequently these species have seen drastic declines and extirpations throughout the region [11,12]. Although tiger parts are worth more than leopard parts, the latter are used as substitutes for tiger parts in traditional Asian medicine given their higher availability [72] or accessibility, thereby causing increased demand and higher prices for leopard parts, especially when tiger numbers decrease. The factors affecting leopard and other wildlife declines in EPL are a microcosm of Southeast Asia, as recent increases in illegal logging in EPL coincided with an explosion in the illegal wildlife trade. Consequently, tiger recently became extirpated in EPL [23] and leopard may be following the same fate. In particular, indiscriminate snaring for the illegal bushmeat market may be the greatest factor causing the rapid decline of leopard in SWS, as blanket snaring for common species such as wild pig and muntjac has been increasing in the area even within protected areas [10]. The increase in snaring has led to the recent documentation of a leopard killed by poachers in SWS [73], suggesting that high rates of poaching of leopard are probably happening throughout SWS, and likely throughout EPL. Establishment of new roads, and improvements to existing roads, during the past 7 years have dramatically increased accessibility to all the protected areas and their core zones in EPL. The SWS is the largest and arguably best protected area in EPL with the lowest level of human disturbance. Additionally, the core zone in SWS contains several ranger stations and is located in the most remote part of the reserve. That leopard density declined so dramatically in the core zone of SWS suggests that leopard numbers elsewhere in EPL might have declined as rapidly, or even more rapidly. In order to save this critical population of leopard, not only do effective law enforcement activities need to increase in SWS and the other protected areas of EPL, but education campaigns are required to decrease the demand for bushmeat, as this leads to indiscriminate snaring across vast areas. In SWS, snaring appeared to have caused a dramatic decline in the leopard population, while the ungulate populations remained stable. This was likely due to the presumably larger home ranges and lower population growth rates of leopard compared to muntjac, wild pig and banteng, thereby causing the leopard population to be more negatively impacted by mortalities from snaring than the ungulate populations.

Contrary to previous studies in Thailand that showed the diet of Indochinese leopard was dominated by relatively small prey, including primates, small ungulates and small carnivores [16–18], the leopard diet in SWS was dominated by large (greater than 30 kg) ungulates (64.3% BC), which did not support our prediction. In Africa, leopard also feed mainly on large ungulates in open habitats [59,74–79]. Leopard may have had similar dietary niche in EPL as the African sites because open habitats typically have a higher carrying capacity for large ungulates than closed forests, resulting in leopard predating more on large ungulates in open habitats due to their higher availability and/or accessibility.

The diameter of confirmed leopard scats in SWS had a considerably wider range (2.1–4.0 cm) than previously reported for leopard in Thailand (2.0–3.0 cm [17]) and Africa (2.0–3.0 cm [80]; 1.9–3.2 [81]). Thus, some leopard scats, particularly those of males, are larger than previously reported. In addition, diameter of leopard scats in our study overlapped the confirmed scat diameters of both larger and smaller sympatric carnivores in Asia, such as tiger, dhole and Asiatic golden cat [82,83]. Therefore, we suggest that genetic analysis should be used in all future studies in Asia that investigate diets of leopard and other sympatric carnivores based on scat analysis.

Our results are the first to record leopard consumption of banteng, which was also the main prey of leopard in SWS (42.2% BC), especially for male leopard (61.5% BC). Adult banteng weigh 600–800 kg [53], and consequently the leopard in SWS are the only known leopard population in the world whose main prey had adult weight greater than 500 kg. Prey species with adult weight greater than 500 kg have been found before in leopard diets in Africa and Asia, but they typically comprise trace amounts of less than 2% of the diet [2]. Wild pig (75–200 kg) also was regularly consumed by leopard in SWS, comprising 22.1% BC, indicating leopard consistently predated on ungulates with some the largest adult weights in SWS. Most previous studies showed that wild pig was avoided by leopard, and consequently wild pig typically comprised only minor parts of leopard diet, presumably because wild pig is relatively large and dangerous prey for leopard [2,45,84]. Only leopard in Golestan National Park (GNP), Iran, was shown to consume higher amounts of wild pig than in our study [85]. Ghouddousi *et al*. [85] concluded high consumption of wild pig by leopard in GNP was not due to a preference for wild pig, but rather due to the high numbers of that prey species on their study site, and the same was likely true in our study (see below).

In addition to ungulates, the next most consumed prey were porcupines, primates and small carnivores (approx. 4% BC each; table 1). Porcupines are relatively dangerous prey for large felids, as both leopard and cougar reportedly have died from injuries from porcupines [86,87]. That 9.6% of leopard scats in SWS contained porcupine quills indicates this species was regularly consumed by leopard. The regular consumption of porcupine could have been due to food stress, as porcupine predation by cougar was shown to increase when primary prey decreased [88]. Alternatively, subadults can prey on porcupines more than adults among large felids [89], indicating it could have been younger leopard that preyed upon porcupine in SWS. Consumption of primates and small carnivores by leopard in SWS was lower than that reported in leopard diets in Thailand [16–18]. The relatively low consumption of primates and small carnivores in SWS may have been due to lower availability compared to Thailand. Alternatively, high consumption of large ungulates by leopard in SWS probably decreased their dependence on smaller prey species compared to Thailand.

Prey selection of leopard in SWS was typical of that found in previous studies, as leopard selectively consumed muntjac, the smallest ungulate species (20–28 kg), which supported our prediction. Leopard consumed wild pig in proportion to availability, and consumed banteng less than expected compared to availability. Thus, our results support previous research that showed preferred prey of leopard is 10–40 kg [2]. Our results indicate the relatively high consumption of banteng and wild pig by leopard in SWS was due to the relatively high density and biomass of these two ungulate species, rather than a preference of these prey species by leopard.

Male and female leopard had remarkably similar diets across prey categories, which supported our prediction. However, male and female leopard had opposite trends with regard to ungulate body size and consumption. Although sample sizes of male and female scats may not have been large enough to determine the full spectrum of their respective diets, we consider it was adequate to demonstrate major differences in consumption patterns between the sexes. In general, male leopard consumed ungulates in proportion to availability, and consequently banteng dominated its diet (figure 4). By contrast, female leopard had a strong positive selection for muntjac, which dominated its diet, and had only a trace amount of banteng. Our results regarding large differences in male and female diets were unexpected, given that previous studies in Africa did not find major differences in body size of ungulates consumed by male and female leopard [59,61]. In one exception, Balme *et al*. [74] found male leopard consumed prey that was on average one-third larger than prey consumed by females. By contrast, our results showed that the main prey consumed by male leopard in SWS was probably 12–30 times larger (depending on age of prey) than the main prey consumed by females. For cougar and cheetah, males were sometimes found to kill larger prey species than did females [20–22], although the difference in prey size was not as great as in our study.

There may have been several reasons for the high consumption of banteng by male leopard in SWS. Firstly, male leopard appeared to be non-selective in their consumption of all the ungulate species, thus the high consumption of banteng was likely related to the high availability of this species in SWS. Secondly, tiger was recently extirpated from EPL [23], and banteng presumably was one of the main prey of tiger in EPL [14]. Therefore, after extirpation of tiger, male leopard may have expanded its dietary niche to include banteng, whose biomass dominated the ungulate community (71.4% of all ungulate biomass available). In SWS, banteng represent an abundant food resource for male leopard, which previously would have been less accessible to it due to competition with the behaviourally dominant tiger. Tiger can kill and displace leopard, and consequently leopard avoid tiger by using suboptimal habitat where there is lower risk of encountering tiger [18,66,90–93]. Leopard also was shown consistently to prefer smaller prey species than did tiger at sites where these species were sympatric, probably as a mechanism to reduce encounters with tiger while hunting [45,48,51]. In the absence of tiger, however, leopard consumed larger prey [50,70], indicating prey size of leopard is affected by tiger presence. In Africa, leopard regularly killed larger ungulate species, including adult common eland (*Taurotragus oryx*) and greater kudu (*Tragelaphus strepsicerso*), in the absence of larger carnivores [1,76], demonstrating the ability of leopard to expand its dietary niche in the absence of larger competitors. Similarly, cougar was found to expand its dietary niche and prey on larger ungulates in the absence of competing large carnivores [94]. Female leopard avoidance of banteng in SWS and preference for smaller and less dangerous species such as muntjac was likely related to its smaller body size compared to males. Interestingly, extirpation of tiger in SWS may have inadvertently allowed leopard sexes to occupy different dietary niches, possibly reducing intersexual competition for food resources.

Dhole also occurred in SWS, although its population was low during our study likely due to a disease outbreak [95]. Even though leopard and dhole potentially competed for food resources, we assumed the dhole population was unlikely to significantly affect the leopard diet and prey selection due to the low numbers of dhole on our study site. Nonetheless, further research is needed to better understand what effects, if any, dhole have on leopard ecology.

Accessible prey, defined as prey consumed both preferentially and in proportion to abundance, for African leopard was recently determined to be 1–45 kg [96]. Our results suggest that male and female leopard can have vastly different accessible prey size ranges, at least under certain conditions. In SWS, male leopard consumed all ungulate species approximately in proportion to their availability, regardless of ungulate size and threat of injury. This indicates the accessible prey range of male leopard in SWS ranged up to 600–800 kg for adult banteng, or up to 300 kg for young banteng, which is 7–13 times larger than that predicted by Clements *et al*. [96]. By contrast, female leopard selectively consumed muntjac the most and wild pig secondarily, both of which were within the accessible prey range reported by Clements *et al*. [96], if we assume females were consuming mostly young wild pig. Additional research is needed to investigate further if male and female leopard have different accessible prey ranges in other areas, as this has important implications regarding their prey requirements and predator–prey relationships.

Although samples from individuals were small, they suggested that three male leopard have different specializations, as banteng ranged from 0 to 72% of their diets, indicating not all males preyed on banteng to the same degree. Although sample size was small for the three individual males, we consider that our results show the potential for males to have different dietary specializations within the same area, similar to that previously reported for large felids. For example, individual specialization on prey species was shown to occur for African leopard [59,76] and cougar [97–99]. Additionally, subadults prey on different species from adults among leopard and cougar [20,21,59,89], leading to individual differences in diet and prey selection that change with age and social status.

Predation impact of leopard in SWS was higher for muntjac than banteng and wild pig, which supported our prediction. That said, predation impact was low for all three ungulate species (less than 4% of populations), which was due to the relatively low leopard density. Regarding threatened ungulate species, leopard consumed only 0.5% of the banteng population, and we did not detect any other threatened ungulates in the leopard diet, suggesting leopard did not negatively impact any threatened ungulate species during the study period. Regarding threatened primates, leopard consumed Colobinae and *Macaca* spp., although in low amounts (1.9% and 1.6% BC, respectively). Although we were unable to distinguish hairs among the macaque species, long-tailed macaque was very common in SWS, and we suspect most consumption of macaque was this species, rather than the threatened macaque species that were rare and possibly absent in the study site. Although we did not determine primate densities, leopard predation likely did not negatively impact any primate populations, primarily because leopard density was relatively low in SWS.

## 5. Conclusion

One of the last remaining potentially viable populations of the Indochinese leopard in Southeast Asia, and the last population in Cambodia, occurs in EPL, and our results show this population is still functioning as part of an important large predator–prey ecosystem. Female leopard fill a typical leopard niche as predator of relatively small-sized ungulates, whereas male leopard appeared to have expanded its dietary niche to include regular predation on banteng, one the largest ungulate species. Consequently, male leopard is filling the predatory niche of the largest carnivore, left open after the extirpation of tiger. However, widespread poaching, which led to the extirpation of the tiger in EPL, now has likely led to a 72% decline of the leopard density in SWS over a 5-year period. Thus, immediate action is needed to prevent the loss of this unique leopard population. Short-term solutions should include increasing the effective enforcement in SWS, such as increasing the number of rangers and anti-poaching units, coinciding with increases in patrols, sweeping areas to remove snares and increasing check points and road blocks to prevent poachers from entering the core zone. Long-term solutions include education campaigns to reduce demand for bushmeat and wildlife parts at the provincial, national and regional levels, as growing demand has led to recent increases in blanket snaring across vast areas [10], including SWS. As a result of improved accessibility to and within the protected areas of EPL, transportation of wildlife products to urban hubs and across international borders is a relatively easy task. Therefore, the strength of transboundary collaborations among relevant bodies needs to be improved to reduce illegal wildlife trade. Other long-term solutions include starting eco-tourism in the area, to generate alternative income for local people, and education programmes highlighting the importance and benefits of protecting the local wildlife. Only with the implementation of these short- and long-term actions will there be any hope for saving the last leopard in Cambodia.

## Supplementary Material

Table S1; Table S2

## Data accessibility

The datasets supporting this article have been uploaded as part of the electronic supplementary material and raw data are available in the Dryad Digital Repository (https://doi.org/10.5061/dryad.4g87s) [100]. The mtDNA sequences were deposited in the Barcode of Life Database (www.boldsystems.org) with accession numbers CBPPA001-17 through CBPPA015-17.
