# Supplementary material for: An adaptable but threatened big cat: density, diet and prey selection of the Indochinese leopard (Panthera pardus delacouri) in eastern Cambodia
Source: R Soc Open Sci. 2018 Feb 7;5(2):171187. doi: 10.1098/rsos.171187 (PMC5830728; doi:10.1098/rsos.171187)
Supplement: Table S1; Table S2 [file rsos171187supp1.docx]

***Rostro-García et al*. An adaptable but threatened big cat: density, diet, and prey selection**

**of the Indochinese leopard (*Panthera pardus delacouri*) in eastern Cambodia**

**Supplementary Tables**

**Table S1.** Summaries from Maximum-likelihood spatially explicit capture-recapture models using different buffer width to estimate leopard density during 2014 in Srepok Wildlife Sanctuary, Cambodia.

| **Buffer width** | **Parameters** | **Value** | **SE** | **LC** | **UC** |
| --- | --- | --- | --- | --- | --- |
| *10 km* |  |  |  |  |  |
|  | *D* | 0.96 | 0.39 | 0.44 | 2.07 |
|  | σ | 3886.15 | 660.92 | 2791.17 | 5410.69 |
|  | G_0_ | 0.01 | 0.01 | 0.01 | 0.03 |
| *20 km* |  |  |  |  |  |
|  | *D* | 0.95 | 0.40 | 0.43 | 2.08 |
|  | σ | 3850.98 | 643.31 | 2781.98 | 5330.76 |
|  | G_0_ | 0.01 | 0.01 | 0.01 | 0.03 |
| *30 km* |  |  |  |  |  |
|  | *D* | 0.95 | 0.40 | 0.43 | 2.08 |
|  | σ | 3850.29 | 643.07 | 2781.66 | 5329.45 |
|  | G_0_ | 0.01 | 0.01 | 0.01 | 0.03 |

| **Parameters** | **Description** |
| --- | --- |
| *D* | Estimated density of leopard per 100 km^2^ |
| G_0_ | Capture probability at home-range centre |
| σ | Function of movement |

**Table S2.** Posterior summaries from Bayesian spatially explicit capture-recapture models used to estimate leopard density during 2014 in Srepok Wildlife Sanctuary, Cambodia.

| **Buffer width** | **Parameters** | **Posterior mean** | **Posterior SD** | **95% Lower HPD Level** | **95% Upper HPD Level** |
| --- | --- | --- | --- | --- | --- |
| *15 km* | *D* | 0.99 | 0.35 | 0.42 | 1.64 |
|  | σ | 4320.00 | 849.00 | 2870.00 | 6030.00 |
|  | λ_0_ | 0.03 | 0.01 | 0.01 | 0.05 |
|  | ψ | 0.25 | 0.09 | 0.09 | 0.43 |
|  | *N* | 21.10 | 7.40 | 9.00 | 35.00 |
| *30 km* | *D* | 1.03 | 0.41 | 0.34 | 1.80 |
|  | σ | 4300.77 | 861.55 | 2907.43 | 6010.96 |
|  | λ_0_ | 0.03 | 0.01 | 0.01 | 0.05 |
|  | ψ | 0.23 | 0.09 | 0.07 | 0.41 |
|  | *N* | 55.59 | 21.83 | 18.00 | 97.00 |

| **Parameters** | **Description** |
| --- | --- |
| *D* | Estimated density of leopard per 100 km^2^ |
| σ | Scale parameter |
| λ_0_ | Baseline encounter rate |
| ψ | Probability that an animal in the augmented data set is a real animal in the space state |
| *N* | Number of individuals in the state-space |
